# Inhibition of G protein-coupled P2Y_2_ receptor induced analgesia in a rat model of trigeminal neuropathic pain

**DOI:** 10.1186/1744-8069-10-21

**Published:** 2014-03-18

**Authors:** Na Li, Zhan-ying Lu, Li-hua Yu, Geoffrey Burnstock, Xiao-ming Deng, Bei Ma

**Affiliations:** 1 Department of Physiology, The Key Laboratory of Molecular Neurobiology, Ministry of Education, Second Military Medical University, Shanghai 200433, P.R. China; 2 Department of Anesthesiology, Changhai hospital, Second Military Medical University, Shanghai 200433, P.R. China; 3 Autonomic Neuroscience Centre, University College Medical School, Royal Free Campus, Rowland Hill Street, London NW3 2PF, UK; 4 Department of Pharmacology and Therapeutics, University of Melbourne, Grattan Street, Parkville, Victoria 3010, Australia

**Keywords:** Antisense oligodeoxynucleotides, ERK, Excitability, I_A_ channels, P2Y_*2*_ receptors, Trigeminal ganglion, Trigeminal neuropathic pain

## Abstract

**Backgrouds:**

ATP and P2X receptors play important roles in the modulation of trigeminal neuropathic pain, while the role of G protein-coupled P2Y_2_ receptors and the underlying mechanisms are less clear. The threshold and frequency of action potentials, fast inactivating transient K^+^ channels (I_A_) are important regulators of membrane excitability in sensory neurons because of its vital role in the control of the spike onset. In this study, pain behavior tests, QT-RT-PCR, immunohistochemical staining, and patch-clamp recording, were used to investigate the role of P2Y_2_ receptors in pain behaviour.

**Results:**

In control rats: 1) UTP, an agonist of P2Y_2_/P2Y_4_ receptors, caused a significant decrease in the mean threshold intensities for evoking action potentials and a striking increase in the mean number of spikes evoked by TG neurons. 2) UTP significantly inhibited I_A_ and the expression of Kv1.4, Kv3.4 and Kv4.2 subunits in TG neurons, which could be reversed by the P2 receptor antagonist suramin and the ERK antagonist U0126. In ION-CCI (chronic constriction injury of infraorbital nerve) rats: 1) mRNA levels of Kv1.4, Kv3.4 and Kv4.2 subunits were significantly decreased, while the protein level of phosphorylated ERK was significantly increased. 2) When blocking P2Y_2_ receptors by suramin or injection of P2Y2R antisense oligodeoxynucleotides both led to a time- and dose-dependent reverse of allodynia in ION-CCI rats. 3) Injection of P2Y_2_ receptor antisense oligodeoxynucleotides induced a pronounced decrease in phosphorylated ERK expression and a significant increase in Kv1.4, Kv3.4 and Kv4.2 subunit expression in trigeminal ganglia.

**Conclusions:**

Our data suggest that inhibition of P2Y_2_ receptors leads to down-regulation of ERK-mediated phosphorylation and increase of the expression of I_A_–related Kv channels in trigeminal ganglion neurons, which might contribute to the clinical treatment of trigeminal neuropathic pain.

## Introduction

Trigeminal neuropathic pain disorders, as typical, atypical, or post-therapeutic trigeminal neuralgias, are pain that is either spontaneous or can be elicited by harmless but crucial activities, such as eating and talking, or by light touch to facial skin [1]. The current treatments do not provide long-lasting relief for these frequently treatment-refractory patients due to a limited understanding of their pathophysiology. Chronic constriction nerve injury (CCI) has characteristics of inflammation and nerve injury [2,3]. Previous studies using a chronic constriction nerve injury model of the infraorbital nerve (ION-CCI) have reported it to be a good model that mimics trigeminal neuralgia of humans [4-7]. The major pathologic changes for trigeminal neuralgia are axonal loss and demyelination in trigeminal root [8]. Constrictive infraorbital nerve injury like constrictive sciatic nerve injury induces demyelination as sources of pathological ectopic firing accompanying mechanical allodynia and heat hyperalgesia [4].

Adenosine 5′-triphosphate (ATP) and uridine 5′-triphosphate (UTP) are released from cells as a consequence of tissue injury and mediate their bio-effects through binding to a large group of cell surface receptors of both P2X or P2Y receptor families [9]. There were early hints that ATP might be involved in pain, including the demonstration of pain produced by injection of ATP into human skin blisters [10,11]. In trigeminal ganglion (TG) neurons, the highly selective distribution of P2X_3_ and P2X_2/3_ receptors within the nociceptive system has suggested a potential role for ATP as a pain mediator [12,13]. Expression of P2Y_1, 2, 4, and 6_ receptors has also been reported in TG neurons [14]. P2Y_2_ receptors are typically expressed on small, nociceptive neurons [15]. *In vitro* studies have demonstrated that co-activation of P2Y_2_ receptors and TRPV channels by ATP could underlie ATP-induced pain [16]. UTP, a selective agonist for P2Y_2_ and P2Y_4_ receptors, activates cutaneous afferent fibers [17], mediates excitation of dorsal root ganglion (DRG) neurons [18] and sensitizes mouse bladder sensory neurons [19]. These results suggest that UTP may be an endogenous nociceptive messenger. However, *in vivo* studies have shown that UTP significantly alleviates mechanical allodynia in a neuropathic pain model [20,21]. However, the effect of activation of P2Y_2_ receptors on neuropathic pain is not clear and requires further study.

Multiple types of voltage-gated ion channels are related to neuronal excitability, such as voltage-gated K^+^ (Kv) channels, which are important regulators of membrane potentials and action potentials in nociceptive sensory neurons [22,23]. In rat small TG neurons, Kv currents have been divided into three types: slow inactivating transient K^+^ current (I_D_), fast inactivating transient K^+^ current (I_A_) and dominant sustained K^+^ current (I_K_) [24]. I_A_ is particularly important in the control of the spike onset, the threshold of the action potential firing, and the firing frequency [25]. Many studies have shown that the Kv1.4, Kv3.4, Kv4.2, and Kv4.3 subunits contribute to the I_A_ channels in DRG neurons [26-28], which suggests that I_A_ has the ability to regulate the neuronal activity of nociceptive neurons. After sciatic nerve injury, the expression of Kv1.4 was decreased in small-diameter DRG neurons [28]. Another study showed that activation with the GABA_B_ receptor agonist baclofen inhibited the excitability of TG neurons, which was mediated by potentiation of both I_A_ and I_K_ in rat small-diameter TG neurons [29]. I_A_, I_K_ and the total K^+^ currents were significantly reduced in rats with inferior alveolar nerve transection and ION-CCI [3,30]. A recent report demonstrated that P2Y_2_ receptors mediate an excitation of DRG neurons through inhibition of K_V_7 channels [18].

In this study, we hypothesize that activation of P2Y_2_ receptors might mediate trigeminal neuropathic pain through regulating the expression and function of Kv1.4, Kv3.4, Kv4.2, and Kv4.3 subunits. We have used pain behavior tests, quantitative reverse transcription–polymerase chain reaction analysis (QT-RT-PCR), immunohistochemical staining and patch-clamp recording to investigate the role of P2Y_2_ receptors in pain behavior, excitability of TG neurons, and modulation of I_A_ channels in rats.

## Materials and methods

### Animals

Experiments were performed on male Sprague–Dawley rats weighing 200–250 g. Rats were kept under standard laboratory conditions with food and water *ad libitum*. They were housed three per cage and maintained on a 12:12 h light: dark schedule at a constant ambient temperature (24 ± 1°C). All experimental procedures were approved by the Institutional Animal Care and Use Committee at the Second Military Medical University.

### Drugs and drug administration

ATP, α,β-methylene-ATP (α,β-meATP), 2-methylthio ADP (2-MesADP), UTP, suramin, U0126 and TEA were purchased from Sigma-Aldrich (St. Louis, MO). Fluoro-Gold was purchased from Biotium (Hayward, CA). For electrophysiology and RT-PCR, ATP, UTP, α,β-meATP, 2-MesADP, suramin and U0126 were dissolved in distilled water to 10 mM, and then diluted to the final concentration (details see the Results). For animal behavioral tests, suramin was diluted in distilled water to 150 μg/50 μl and 15 μg/50 μl and injected only once per dilution, respectively. P2Y_2_ receptor antisense oligodeoxynucleotides (AS-ODN) was dissolved in water to 15 μg/50 μl and usually injected every 12 h within a 48 h period.

For animal behavioral tests, we performed a peripheral target injection to the TG via the infraorbital foramen as described previously by Neubert [31]. Briefly, at day 9 after surgery, rats were anaesthetized with diethyl ether. A sterile stainless steel needle was inserted medial (1–2 mm) to the palpated portion of the zygomatic process through the infraorbital foramen. The needle was positioned at ~10° angle relative to the midline of the head. The tip of the needle was advanced approximately 20 mm along the infraorbital canal and subsequently through the foramen rotundum, then the corresponding drugs were injected. The mechanical pain threshold was then determined every 5 min or 12 h after injection.

### P2Y_2_ receptor antisense oligodeoxynucleotides (AS-ODN)

P2Y_2_ receptor AS-ODN was purchased from Invitrogen Company. Oligonucleotides to rats P2Y_2_ were synthesized and purified by Integrated DNA Technologies (ADT). The sequence was as follow: antisense 5′-CCAGGAGTCCAGGCCTGCTGCCATTGCC-3′. The sequences were checked for uniqueness using the National Center for Biotechnology Information’s Local Alignment Searchtool (BLAST) based on a previous study [32].

### Surgery and behavior test

#### Chronic constriction injury of the infraorbital branch of trigeminal nerve (ION-CCI) and sham surgery

Rats underwent CCI of the right ION as previously described [3]. Briefly, rats were anaesthetized with sodium pentobarbital (50 mg/kg i.p.) and a small incision (approximately 5 mm) was made at the juncture between the zygomatic arch and nasal bone, under the right eye. The muscle was dissected to exposure the infraorbital branch of the TG nerve until it was clearly visible through the incision and two ligatures (4–0 chromic catgut) were loosely tied (with about 2 mm spacing) around it. The loose ligature method was used according to the procedure developed by Imamura et al. [33]: the ligatures reduced the diameter of the nerve by a just noticeable amount and retarded, but did not interrupt the circulation through the superficial vasculature. The incision was sutured with 1.0 silk. Sham-operated rats were treated identically, but no ligatures were applied to the ION. All operations were performed aseptically. After surgery, all rats were maintained in a warm room until they recovered from anesthesia and no antibiotics were administered.

### Behavior test

Rats were allowed to acclimate for at least 3 days before use in experiments. All the experiments were carried out between 9:00 and 14:00 in the animal housing room. Before each testing session, animals were placed in individual plastic cages and left to adapt to the environment for at least 15 min. The mechanical pain threshold was tested one day before and every three days after surgery in the two groups. In accordance with our previous study, we injected drugs and performed behavior tests at day 9 after surgery when the mechanical pain threshold of rats was lowest. Each rat received drugs only once and was used in only one experiment. The mechanical pain threshold (PWT) was determined with a rigid von Frey filament coupled with a force transducer (Electrovonfrey, model no: 2391, IITC Inc. Woodland Hills, CA), as previously described [34]. Stimuli were applied within the ION territory, around the center of the vibrissal pad, on the hairy skin surrounding the mystacial vibrissae, ten consecutive times with 2 s each time at 30-sec intervals on the nerve-injured side [35]. The PWT was considered as the lowest force of the filaments that produced a brisk head withdrawal, touching or scratching the facial regions upon mechanical stimulation [35]. The mechanical pain threshold were measured every 5 min or 12 h following drug administration. The persons conducting the behavioral measurements were blind to the treatments.

### Retrograde labeling of TG neurons innervating the facial skin

TG neurons innervating the facial skin were identified for electrophysiology and immunohistochemistry using the fluorogold (FG)-labeling method [36]. A FG solution (2% in distilled water, 50 μl) was injected into the facial skin in the bilateral region of the whisker pad using a 31-gauge microsyringe.

### Cell culture

Control male Sprague–Dawley rats were used for patch-clamp recording and RT-PCR analysis. Rats were decapitated and bilateral TG were rapidly removed, de-sheathed, cut and incubated in 2 ml Ca^2+^- and Mg^2+^-free Hanks’ balanced salt solution with 10 mM HEPES buffer (pH 7.4) (HBSS; Life Technologies) containing 1.5 mg/ml collagenase (Class II, Worthington Biochemical Corporation, UK) and 6 mg/ml bovine serum albumin (Sigma Chemical Co., Poole, UK) at 37°C in a shaking bath (170 rpm) for 30 min. This was followed by incubation in 2 ml HBSS containing 1 mg/ml trypsin (Sigma) at 37°C in a shaking bath (170 rpm) for 10 min. The solution was replaced with 1 ml growth medium comprising L-15 medium supplemented with 10% bovine serum, 50 ng/ml nerve growth factor, 0.2% NaHCO_3_, 5.5 mg/ml glucose, 200 i.u./ml penicillin and 2 g/ml streptomycin. The ganglia were dissociated into single neurons by gentle mechanical trituration and plated onto 35 mm Petri dishes coated with 10 μg/ml laminin (Sigma) [37]. Cells were maintained at 37°C in a humidified atmosphere containing 5% CO_2_. All neurons were studied after 16 h removal from the animals. For RT-PCR, cultured TG neurons were incubated in growth medium without 10% bovine serum, in order to eliminate glia cells.

### Real-time quantitative reverse transcription–polymerase chain reaction analysis

#### RNA extraction and RT-PCR

Total RNA was extracted using an RNeasy Mini Kit (QIAGEN; Clifton Hill, Australia). RNA purity was determined using a method of ultraviolet spectrophotometry at a wavelength of 260–280 nm. 2 μg of total RNA was reversely transcribed to complementary DNA in a 20 μl reaction mixture containing 1× reverse transcriptase buffer (15 mM MgCl_2_, 375 mM KCl, 50 mM DTT, 250 mM Tris–HCl, pH 8.3), 10 mM dNTP, 20 U RNase inhibitor, 200 U M-MLV reverse transcriptase, and 50 ng of oligo (deoxythymidine)_15_ primer. Reaction time was at least 1 h at 42°C. The cDNA was stored at −20°C until real-time polymerase chain reaction (RT-PCR). All reagents, with the exception of the RNeasy Mini Kit, were from Promega Corp. (Madison, WI).

### Real-time RT-PCR

Quantitative RT-PCR amplification was performed with SYBRGreen (Applied Biosystems; Scoresby, Australia) using Roto-gene RG3000 (Australia) in a 20 μl reaction mixture. The solution consisted of 1.0 μl diluted RT-PCR product, 0.25 μM of each of the paired primers, and 10 μl real-time PCR SYBR Green Master Mix (QIAGEN, Clifton Hill, Australia). RNA levels were measured with specifically designed primers. That for Kv1.4 was: 5′-TTG TGA ACG CGT GGT AAT AAA TGT GT-3′ (forward), 5′-GGC GGC CTC CTG ACT GGT AAT AAT A-3′ (reverse); for Kv3.4: 5′-CCA CGG GGC AAT GAC CAC ACC-3′ (forward), 5′-ACA CAG CGC ACC CAC CAG CAT TCC T-3′ (reverse); for Kv4.2: 5′-GCC GCA GCG CCT AGT CGT TAC C-3′ (forward), 5′-TGA TAG CCA TTG TGA GGG AAA AGA GCA-3′ (reverse); and for Kv4.3: 5′-CTC CCT AAG CGG CGT CCT GGT CAT T-3′ (forward), 5′-CTT CTG TGC CCT GCG TTT ATC TGC TCT C-3′ (reverse) against the sequences downloaded from Genbank (accession no. X90651, 708–731 and 1126–1147). The PCR condition was 95°C for 2 min, followed by 40 cycles of 95°C, 20 s; 65°C, 25 s; 72°C, 25 s for Kv1.4, Kv3.4, Kv4.2, Kv4.3 and was 95°C for 2 min, followed by 40 cycles of 95°C, 20 s; 63°C, 25 s; 72°C. RT-PCR for the housekeeping gene β-actin was performed for each sample. The primer for amplification of β-actin was: ATGGTGGGTATGGGTCAGAAGG (forward); TGGCTGGGGTGTTGAAGGTC (reverse). The absolute mRNA level of target gene in each sample was calculated using a standard curve and then by the ratio to β-actin in each sample. The specificity of the primers was verified by examining the melting curve as well as sequencing of the QT-RT-PCR products. The melting curve of QT-RT-PCR showed a single sharp peak for Kv1.4, Kv3.4, Kv4.2, Kv4.3 and β-actin PCR products. The lengths of PCR products of Kv1.4, Kv3.4, Kv4.2, Kv4.3 and β-actin mRNA were 199, 134, 261, 108 bp and 265 bp, respectively.

### Whole-cell patch clamp recording

Whole-cell patch-clamp recording was undertaken at room temperature with an Axopatch 200B amplifier (Axon Instruments, Foster City, CA, USA). Membrane potential held at −60 mV, signals were filtered at 2 kHz (−3 dB frequency, Bessel filter, 80 dB per decade), then digitized at 10–50 kHz (Digidata 1320A interface, Axon Instruments). The leak current was subtracted from the potassium currents using Clampfit programs. Patch electrodes had resistance of 2–5 MΩ. For voltage-recordings, the pipette solution contained the following (in mM): K gluconate 120, KCl 10, NaCl 5, MgCl_2_•6H_2_O 2, CaCl_2_•2H_2_O 1, HEPES 10, EGTA 11, Mg-ATP 2, Li-GTP 1 (pH adjusted to 7.4 with KOH). The external solution contained (in mM): NaCl 145, KCl 3, CaCl_2_•2H_2_O 1, MgCl_2_•6H_2_O 2, HEPES 10, glucose 10, (pH adjusted to 7.4 with NaOH). Test solutions bathing the cytoplasmic face of the patch membrane contained (in mM): NMDG 145, TEA 25, KCl 3, MgCl_2_•6H_2_O 0.6, CdCl_2_ 1, CaCl_2_•2H_2_O 2.5, HEPES 10, glucose 10 (pH adjusted to 7.4 with tris-base and 300 mOsM). CdCl_2_ was included to block voltage-gated calcium channels. NMDG and TEA were included to reduce currents from voltage-gated sodium channels, I_K_ currents, hyperpolarization-activated cation channels, and capsaicin-induced inward currents [27,38]. A protocol was used as previous described [39], briefly, a pre-pulse (−120 mV, 100 ms) was followed by test pulses (400 ms) from −60 to +60 mV with 10 mV increments, and only those cells that exhibited minimal outward currents during the pre-pulse were analyzed. For current-recordings, action potentials were recorded under current-recordings. During a 400-ms injection of a positive current (ranging from −40 to 450 pA), a single action potential could be evoked, depending on the type of neuron (Aβ-, Aδ- and C-units) [3], for example Aδ-units were frequently encountered at a later period after ION-CCI. Cultured TG neurons with soma diameters ranging from 18 to 39 μm were used for action potential recording, for they are consistent with nociceptive Aδ- and C-neurons [39]. Those neurons with retrograde labelling were used for I_A_ recording. The amplitude of the I_A_ was measured at the peak. Whole-cell current–voltage (I-V) curves for individual neurons were generated by calculating the peak outward current at each testing potential and normalizing to the cell capacitance.

### Western blotting analysis

TG were harvested and homogenized in cold lysis buffer (20 mM Hepes buffer, pH 7.4, 10 mM KCl, 1.5 mM MgCl_2_, 1 mM EDTA, 1 mM EGTA, 1 mM DTT, 0.1 mM PMSF, 5 mg/mL pepstatin A, 10 mg/mL leupeptin and 10 mg/mL aprotinin) using a Dounce homogenizer. Protein concentration was determined with a bicinchoninic acid (BCA) assay kit using bovine serum albumin as a standard (Pierce Biotechnology, Inc., Rockford, IL) and then heated to 95°C. Proteins were separated using sodium dodecyl sulfate polyacrylamide gel electrophoresis (SDS-PAGE) on 12% Tris–HCl gels (BioRad, Hercules, CA) and electrophoretically transferred to polyvinylidene difluoride membranes (Bio-Rad Laboratories, USA) at 120 V for one and half an hour in Towbin buffer, pH 8.3, to which 20% (V/V) methanol had been added. After transfer, the membranes were blocked with 5% (mass/vol) non-fat dried milk in Tri-buffered saline containing 0.05% Tween 20 (TBST) for 1 hour, then incubated with the primary antibodies: P2Y_2_ (rabbit anti-rat polyclonal, IgG 1:500, Santa Cruz Biotechnology, Santa Cruz, CA) or ERK (rabbit anti-rat polyclonal, IgG 1:1000, Cell Signaling) and β-actin (mouse monoclonal, IgG 1:8000, Sigma, USA). After three washes with TBST, the membranes were incubated with the secondary antibody (goat anti-rabbit polyclonal, IgG 1:8000, Santa Cruz Biotechnology, Santa Cruz, CA). Membranes were rinsed at room temperature in Tris-buffered saline containing Tween 20 (TBST) followed by TBS 3 times and visualized using an Odyssey Infrared Imaging System (LICOR, Lincoln, NE). Densitometric quantification of the P2Y_2_, ERK and β-actin protein bands of the Western blot were determined using Odyssey software version 1.0 (LI-COR, Lincoln, NE) and expressed as a relative ratio of P2Y_2_/β-actin and ERK/β-actin.

### Immunofluorescence histochemistry

Rats were anaesthetized with sodium pentobarbital (50 mg/kg i.p.) and perfused transcardially with 0.1 M phosphate buffer solution (PBS), pH 7.4 and subsequently with fresh 4% paraformaldehyde phosphate buffer (PB) solution. After the perfusion, TGs were harvested and fixed in 4% paraformaldehyde for 4 hours. They were then transferred into 20% sucrose for at least 3 days. Series frozen transverse sections (10 μm thick) were made through the TG with a cryostat (Leica, CM1850, Germany), collected and then washed 3 × 5 min in cold PBS. The preparations were then preincubated in antiserum solution 1 (10% normal bovine serum, 0.2% Triton X-100, 0.4% sodium azide in 0.01 mol/l PBS pH 7.2) for 30 min. For double-immunostaining of P2Y_2_ and Kv1.4 or Kv3.4 or Kv4.2 or Kv4.3, sections were incubated in a mixture of rabbit polyclonal P2Y_2_ (1:50 dilution, Santa Cruz Biotechnology, Santa Cruz, CA, USA) and mouse monoclonal Kv1.4 (1:200 dilution, Abcam, HongKong, China) or goat polyclonal KCNC4 (KV3.4) (1:100 dilution, Santa Cruz Biotechnology, Santa Cruz, CA, USA) or goat polyclonal Kv4.2 (1:50 dilution, Santa Cruz Biotechnology, Santa Cruz, CA, USA) or goat polyclonal Kv4.3 (1:50 dilution, Santa Cruz Biotechnology, Santa Cruz, CA, USA) at 4°C overnight. The sections were subsequently incubated with FITC-conjugated affinipure donkey anti-rabbit IgG (1:200 dilution, Jackson ImmunoResearch Laboratories, West Grove, PA, USA) for 1 h to visualize P2Y_2_ receptors, fluorophore-labeled donkey anti-mouse IgG (1:1500 dilution, Invitrogen life technologies, Grand Island, NY, USA) for 1 h to visualize Kv1.4, Cy3-conjugated affinipure donkey anti-goat IgG (1:200 dilution, Jackson ImmunoResearch Laboratories, West Grove, PA, USA) for 1 h to visualize Kv3.4, Cy3-conjugated affinipure donkey anti-goat IgG (1:300 dilution, Jackson ImmunoResearch Laboratories, West Grove, PA, USA) for 1 h to visualize Kv4.2 or Kv4.3, respectively. All staining procedures were carried out at room temperature and all the incubations were separated by three washes in PBS, 5 min each. The immunoreactivity was visualized by fluorescence microscopy.

### Statistical analysis

All data are presented as means ± SEM. The electrophysiological data were analyzed using the clampfit 9.0 and origin 7.0. For current-clamp recording, differences between the means of action potentials were tested for significance using unpaired Student’s t-tests. For voltage-clamp recording and animal behavior test, differences between the means were tested for significance using repeated measures ANOVA followed by Dunnett’s analysis. For RT-PCR results, differences among groups were tested for significance using two way ANOVA followed by Dunnett’s analysis. For Western-blot and immunofluorescence histochemical results, differences among groups were tested for one-way ANOVA followed by Tukey’s HSD and unpaired Student’s t-tests. Differences were considered as statistically significant when the p value was lower than 0.05 (*p* < 0.05).

## Results

### Effects of P2Y_2_ receptors on Kv1.4, Kv3.4, Kv4.2 and Kv4.3 in control rat TG neurons

Double-immunofluorescence staining for P2Y_2_ receptors and Kv1.4 or Kv3.4 or Kv4.2 or Kv4.3 was performed on TG neurons in sham and ION-CCI groups. In the sham group, 272 out of 301 (90.4%) P2Y_2_ receptor-positive neurons were Kv1.4 positive; 302 out of 307 (98.1%) P2Y_2_ receptor-positive neurons were Kv3.4 positive; 274 out of 325 (84.3%) P2Y_2_ receptor-positive neurons were Kv4.2 positive; and 159 out of 201 (79.1%) P2Y_2_ receptor-positive neurons were Kv4.3 positive. Most of them had soma diameters ranging from 25 to 34 μm (n = 294 neurons).

#### UTP induces hyperalgesia in control rats

**Figure 1:**
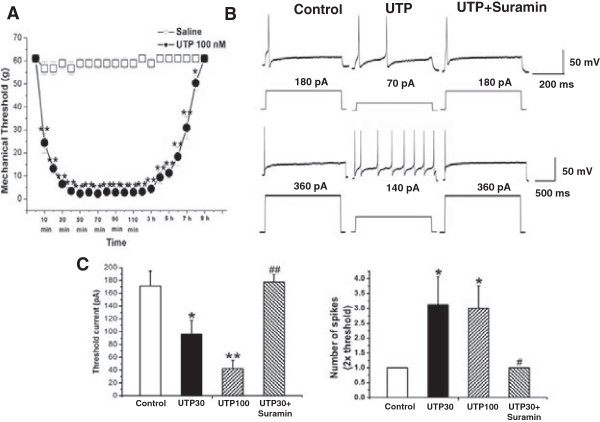
**UTP induced hyperalgesia and enhanced the excitability of small-diameter TG neurons in control rats. (A)** Changes in rat facial mechanical pain threshold after injection of UTP or saline. UTP (100 nM, 50 μl) significantly decreased the mechanical pain threshold of the whisker pad 20 min after injection and remained for at least 3 h, which then declined 9 h after injection. n = 8 for each group, **, *p* < 0.01 vs saline. **(B)** Original traces of action potentials during intracellular current injection in control TG neurons. **(C)** Mean threshold currents (left panel) and mean number of spikes (right panel) in the presence, absence of UTP (30,100 μM) treatment and UTP (30 μM) co-application with suramin (100 μM) for 16 h. The depolarizing step current amplitude is twice as much as threshold. n = 8 neurons, *, *p* < 0.05, **, *p* < 0.01 vs control; #, *p* < 0.05, ##, *p* < 0.01 vs UTP30.

We investigated the role of UTP, an agonist of P2Y_2_ and P2Y_4_ receptors on control rats. UTP (100 nM, 50 μl) significantly decreased the mechanical pain threshold of the whisker pad 20 min after injection and this remained at least for 3 h (Figure 1A, n = 8 for each group, *p* < 0.01); it then declined 9 h after the injection (Figure 1A).

#### UTP enhances the excitability of small-diameter TG neurons in control rats

Based on the results of the animal behavioral test, we explored the effect of P2Y_2_ receptor activation on the electrophysiological properties of small-diameter TG neurons of rats. Action potentials generated in these TG neurons by square-pulse stimulation are illustrated in Figure 1B. Incubation of TG neurons from control rats with UTP 30 or 100 μM for 16 h, caused dose-dependent decrease in the mean threshold intensities for evoking action potentials (UTP30 group: 96.3 ± 21.2 pA; UTP100 group: 42.5 ± 13.1 pA; control group: 171.3 ± 23.2 pA; n = 8, Figure 1B,C, *p* < 0.05 or *p* < 0.01 vs control). The decrease of mean threshold intensities for evoking action potentials by UTP 30 μM was reversed by co-incubated with suramin 100 μM (UTP30 group: 96.3 ± 21.2 pA; suramin group: 177.5 ± 11.8 pA; n = 8, Figure 1B,C, *p* < 0.05). As shown in Figure 1C, the mean number of spikes evoked in the UTP-incubated TG neurons during depolarizing step pulses at 2 × threshold was significantly higher in the UTP-incubated TG neurons than those in the control neurons but did not show any dose-dependent changes (control: 1 spikes/400 ms,; UTP-incubated TG neurons (30 or 100 μM): 3.1 ± 0.9 spikes/400 ms or 3.0 ± 0.8 spikes/400 ms; n = 8, *p* < 0.05). The increase of mean number of spikes by UTP 30 μM was blocked by co-incubated with suramin 100 μM (Figure 1B,C, n = 8, *p* < 0.05 vs UTP 30 group).

#### Activation of P2Y_2_ receptors mediates a functional inhibition of I_A_ channels by UTP in FG-labeled small-diameter TG neurons in control rats

**Figure 2:**
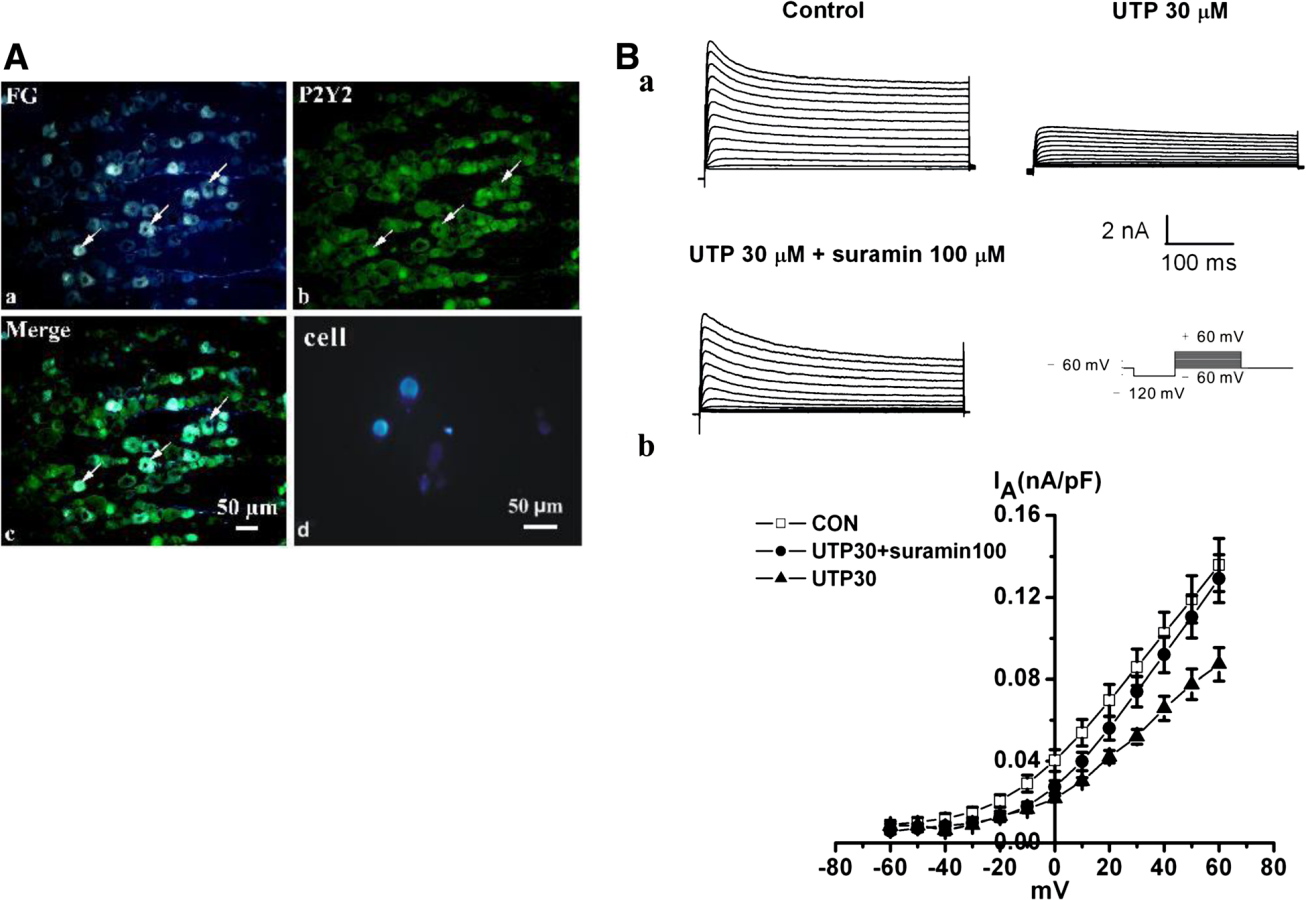
**Effect of UTP and suramin on I**_**A **_**of small-diameter and FG-labeled TG neurons, with diameters ranging from 18 to 39 μm in control rats. (A)** Fluorescence microscopic view of TG neurons from control rats. (a) Retrograde labeling of TG neurons (blue) innervating whisker pad skin. (b) P2Y_2_ receptor-positive (green) TG neurons were seen in the section of TG. (c) The merged images (purple) of retrograde labeling of TG neurons and P2Y_2_ receptor-positive TG neurons from the same section, indicating co-localization. (d) Retrograde labeling of TG neurons (blue) innervating whisker pad skin in cultured TG neurons. **(B)** Electrophysiology recording for small-diameter and FG-labeled TG neurons in control rats. (a) Representative traces showing that the application of 30 μM UTP reduced I_A_. Suppression of the mean peak amplitudes of I_*A*_ seen after UTP application was antagonized by suramin 100 μM. (b) Current–voltage relationship for the effects of UTP and suramin on I_A_. Each value represents the mean ± SEM (con: 0.14 ± 0.01 nA, n = 9; UTP: 0.09 ± 0.01 nA, n = 20, *p* < 0.05 vs control; suramin: 0.13 ± 0.01 nA, n = 9). I_A_ was initiated via a prepulse (100 ms) of −120 mV and test pulses (400 ms) from −60 to +60 mV in a 10 mV step.

FG-labeled TG neurons are illustrated in Figure 2A. We observed whether activation of P2Y_2_ receptors could functionally inhibit I_A_ subunits in these TG neurons. For voltage-clamp experiments, typical waveforms of depolarization-activated I_A_ are shown in Figure 2B. After incubation with UTP (30 μM) for 16 h, the mean peak amplitude of I_A_ was significantly suppressed compared with that of control (0.12 ± 0.01 nA vs 0.06 ± 0.01 nA, n = 12, *p* < 0.01). The suppression of peak amplitudes of I_A_ by UTP (30 μM) was then blocked by co-application of suramin (Figure 2B, con: 0.14 ± 0.01 nA, n = 9; UTP: 0.09 ± 0.01 nA, n = 20, *p* < 0.05; suramin: 0.13 ± 0.01 nA, n = 9, *p* < 0.01). We did not see any dose-dependent changes in I_A_ when using UTP 100 μM (0.08 ± 0.01 nA, n = 20).

**Figure 3:**
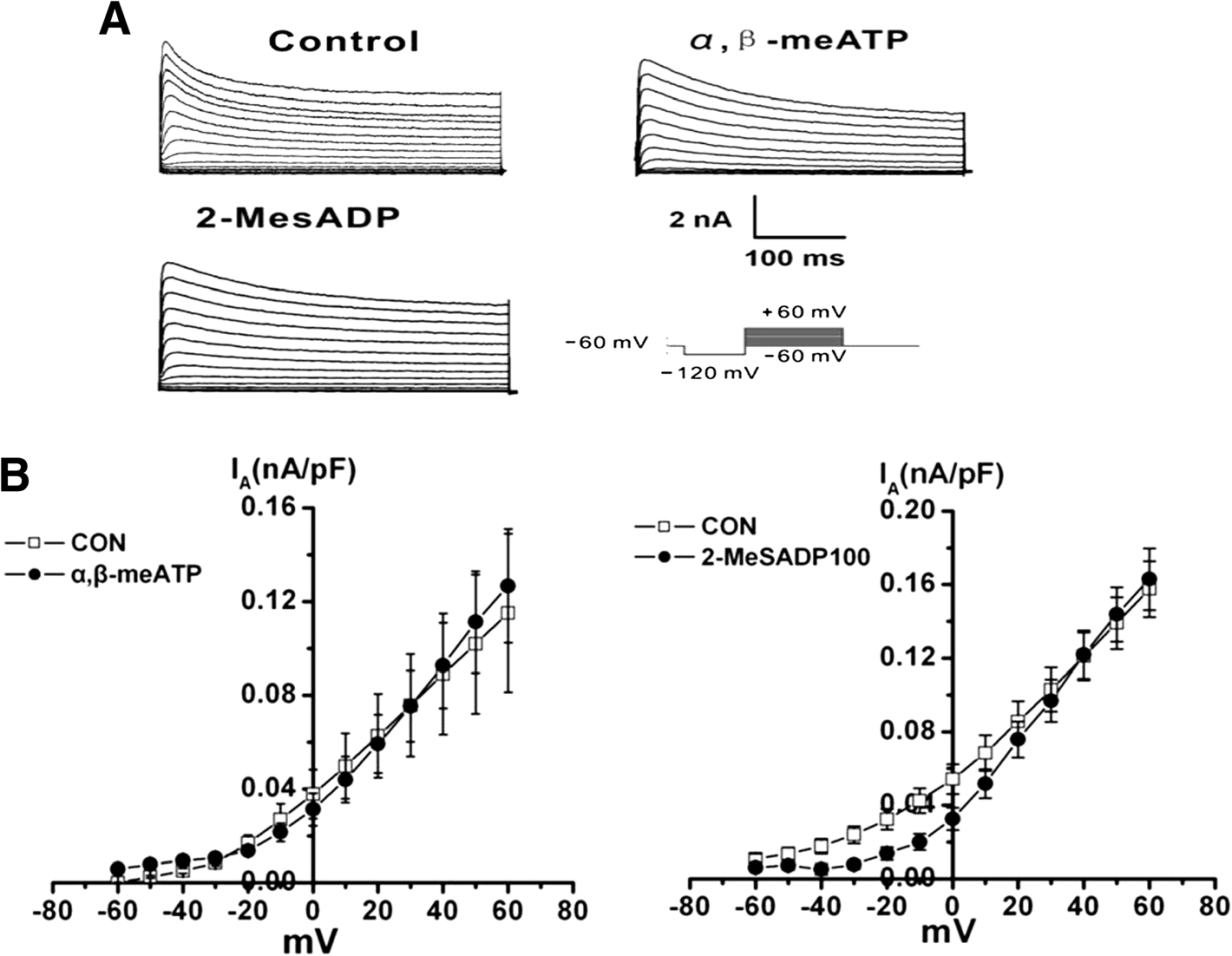
**Effect of α,β-meATP and 2-MeSADP on I**_**A **_**of small-diameter TG neurons in control rats. (A)** I_A_ was initiated via a prepulse (100 ms) of −120 mV and test pulses (400 ms) from −60 to +60 mV in a 10 mV step. Original traces showing that the application of 10 μM α,β-meATP and 100 μM 2-MeSADP for 16 h did not suppress I_A_. **(B)** Current–voltage relationship for both on I_A_. Each value represents the mean ± SEM (Con: 0.14 ± 0.01 nA, n = 20; α,β-meATP: 0.13 ± 0.02 nA, 2-MeSADP: 0.13 ± 0.02 nA, n =11, *p* > 0.05).

In order to observe whether other pain-related P2 receptors were involved in the inhibition of I_A_, α,β-meATP, a P2X_3_ and P2X_2/3_ receptor agonist, and 2-MeSADP, a P2Y_1_ receptor agonist, were used. We did not find any changes in I_A_ following application of either α,β-meATP or 2-MeSADP, respectively (Figure 3). This implied that P2X_1_, P2X_3_, P2Y_1_, P2Y_12_ and P2Y_13_ receptors were not involved.

#### UTP-induced reduction in the expression levels of I_A_ subunits (Kv1.4 or Kv3.4 or Kv4.2 and Kv4.3) in control TG neurons via P2Y_2_ receptors

**Figure 4:**
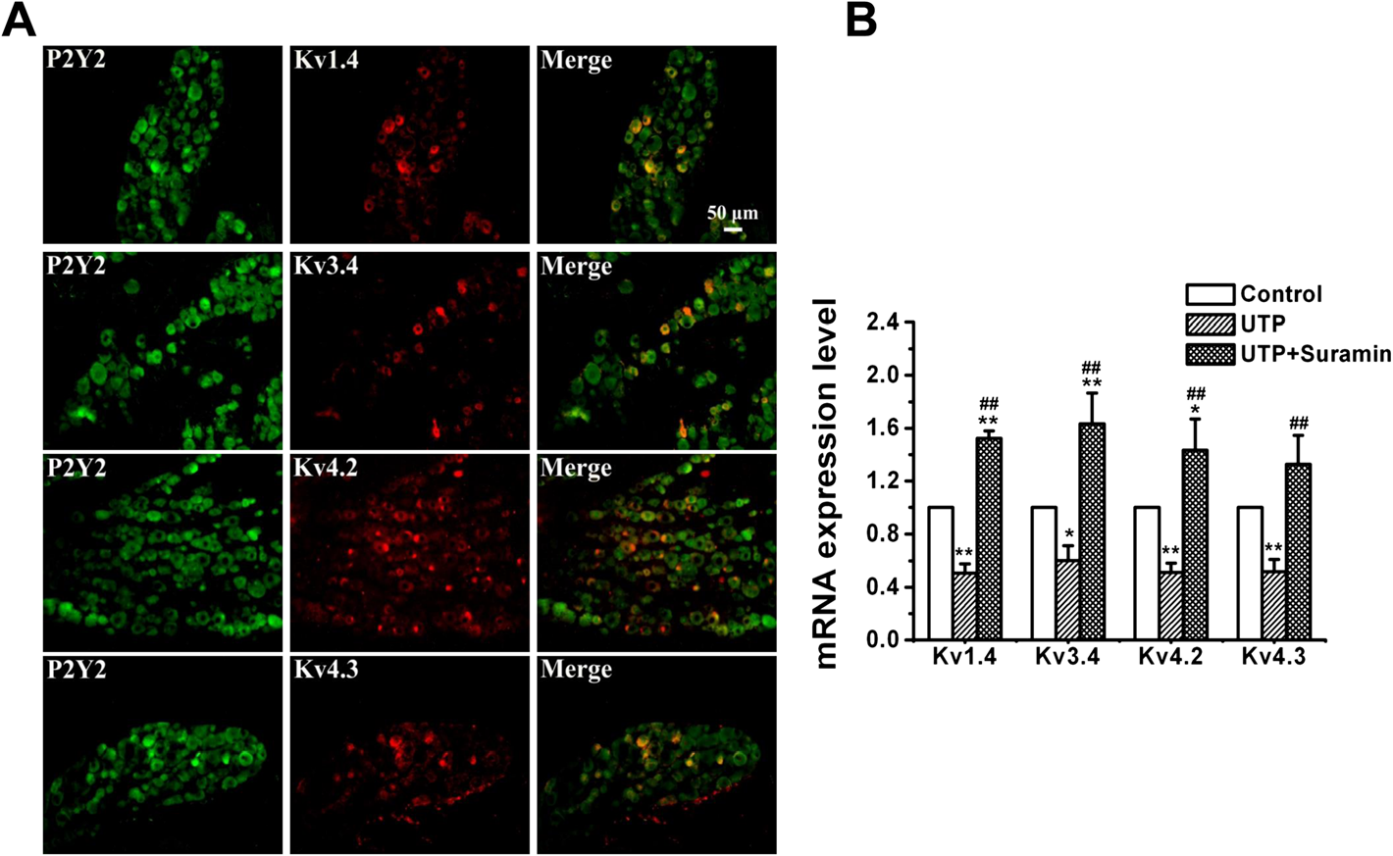
**Effect of UTP on the expression levels of I**_**A **_**subunits in control TG neurons. (A)** Double-immunostaining revealed the expression of Kv1.4, Kv3.4, Kv4.2 and Kv4.3 subunits in P2Y_2_ receptor-positive neurons in the ION-CCI TG sections. The P2Y_2_ receptor-positive TG neurons also expressed Kv1.4, Kv3.4, Kv4.2 and Kv4.3, respectively, n = 5 rats. **(B)** Reduction in the mRNA levels of I_A_ subunits by UTP in cultured TG neurons from control rats. Treatment with suramin (100 μM) in the UTP-incubated (30 μM) TG neurons for 16 h in control rats reversed the decrease in the mRNA levels of Kv1.4, Kv3.4, Kv4.2, and Kv4.3 subunits. n = 10 samples in each group, *, *p* < 0.05, **, *p* < 0.01 vs control; ^##^, *p* < 0.01 vs UTP.

Firstly, we performed double immunofluorescent staining for P2Y_2_ receptors and Kv1.4 or Kv3.4 or Kv4.2 or Kv4.3 on TG neurons in rats, respectively. The results showed that the P2Y_2_ receptor-positive TG neurons also expressed Kv1.4, Kv3.4, Kv4.2 and Kv4.3 (Figure 4A, n = 5 rats), respectively. We further found that UTP induced a significant decrease in the expression of Kv1.4, Kv3.4, Kv4.2, and Kv4.3 mRNA in TG (Figure 4B, n = 10 samples in each group, *p* < 0.01, *p* < 0.05, *p* < 0.01, *p* < 0.01 *vs* sham group). Treatment with suramin (100 μM) in the UTP (30 μM)-incubated TG neurons for 16 h in control rats reversed the decrease of the expression of Kv1.4, Kv3.4, Kv4.2, and Kv4.3 mRNA (Figure 4B, n = 10 samples in each group, *p* < 0.01, *p* < 0.01, *p* < 0.01, *p* < 0.01 *vs* UTP group).

### Effects of P2Y_2_ receptors on Kv1.4, Kv3.4, Kv4.2 and Kv4.3 in ION-CCI rat TG neurons

#### The role of P2Y_2_ receptors on mechanical allodynia in ION-CCI rats

**Figure 5:**
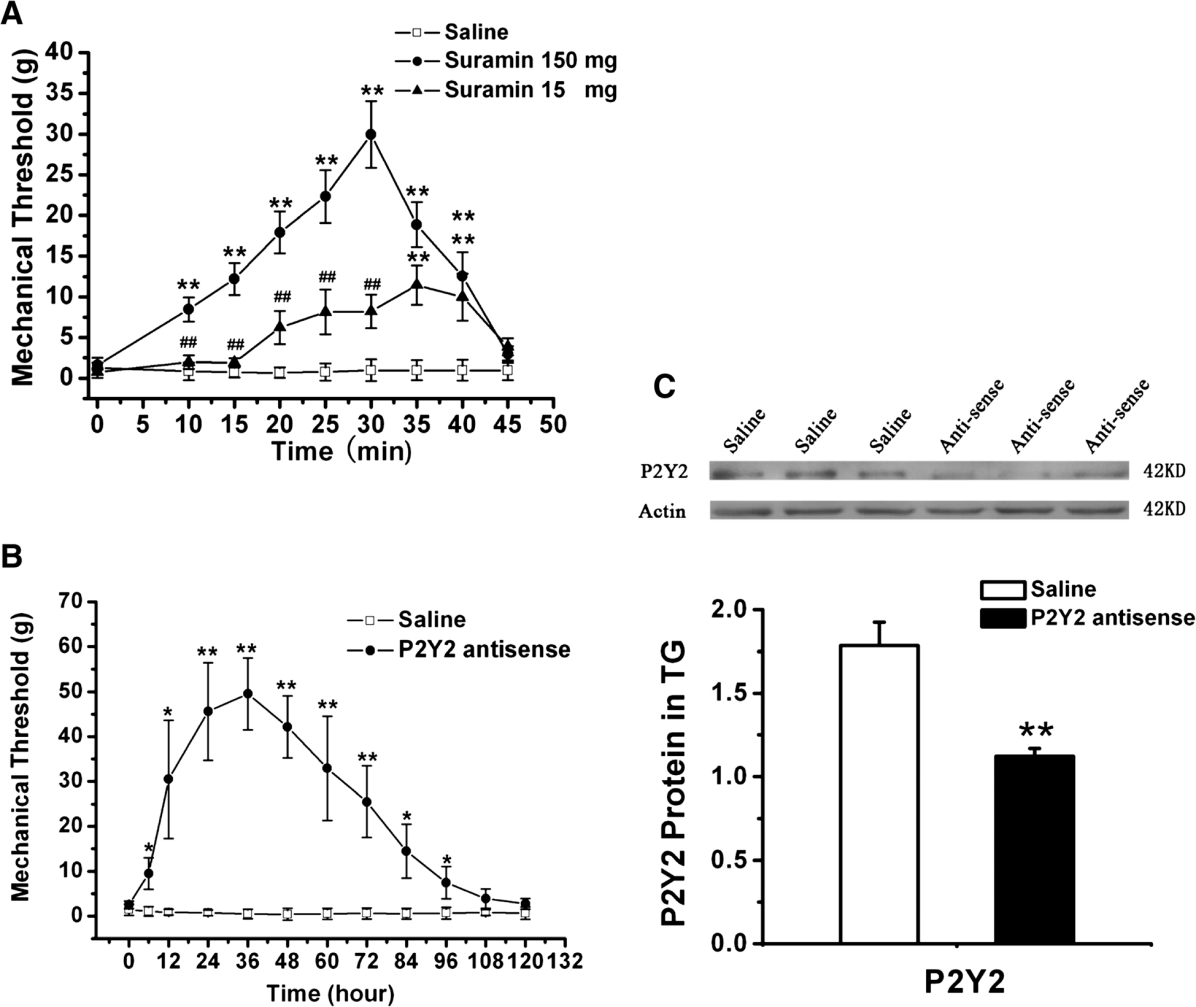
**The role of P2Y**_**2 **_**receptors in mechanical hyperalgesia in ION-CCI rats. (A)** The peripheral target injection to TG of suramin (0.3-3 μg/μl) reduced mechanical allodynia in the whisker pad. n = 6-8, **, *p* < 0.01 compared with injection of saline, ^##^, *p* < 0.01 compared with injection of high-dose suramin. Suramin led to a time- and dose-dependent increase in PWT, this anti-allodynia effect started 10 min after the suramin injection and remained for at least 45 min. **(B)** The peripheral target injection to TG of P2Y_2_ antisense oligodeoxynucleotides significantly alleviated mechanical allodynia of the whisker pad. n = 5, *, *p* < 0.05, **, *p* < 0.01 compared with injection of saline. The effect started at 6 h and persisted for at least 120 h. **(C)** Western blots showed successful suppression of P2Y_2_ receptor expression in TG by P2Y_2_ receptor antisense oligodeoxynucleotides treatment n = 4 for each group, **, *p* < 0.01.

The effects of suramin on the mechanical pain threshold of ION-CCI rats were determined. As shown in Figure 5A, suramin (15,150 mg) led to a time- and dose-dependent increase in PWT (saline: 0.96 ± 0.5 g, n = 8; suramin 15 mg: 11.45 ± 2.4 g, n = 6 rats; suramin 150 mg: 29.96 ± 4.1 g, n = 7) compared with that of control (ION-CCI) rats (*p* <0.01). This anti-allodynia effect started 10 min after the suramin injection and remained at least 45 min. Further, we injected P2Y_2_ receptor AS-ODN twice a day for 2 days through the peripheral target injection to TG via the infraorbital foramen and then determined whether it could improve neuropathic pain 9 days after injection. The PWT of whisker pad was significantly increased after injection of P2Y_2_ receptor AS-ODN, compared with that of the control (ION-CCI) rats (Figure 5B, *p* < 0.05). The effect started at 6 h and persisted for at least 120 h (Figure 5B, saline: 0.49 ± 0.1 g, n = 7 rats; AS-ODN: 49.54 ± 8.0 g, n = 5). To confirm that P2Y_2_ receptor AS-ODN had knocked down the expression of P2Y_2_ receptor, the expression of P2Y_2_ receptor after P2Y_2_ receptor AS-ODN injection was investigated. Compared with that in the saline group, injection of P2Y_2_ receptor AS-ODN significantly reduced P2Y_2_ receptor protein expression (n = 4, *p* < 0.01, Figure 5C).

#### P2Y_2_ receptor antisense reverses the decreased expression of Kv1.4 or Kv3.4 or Kv4.2 or Kv4.3 on P2Y_2_-positive TG neurons after ION-CCI

**Figure 6:**
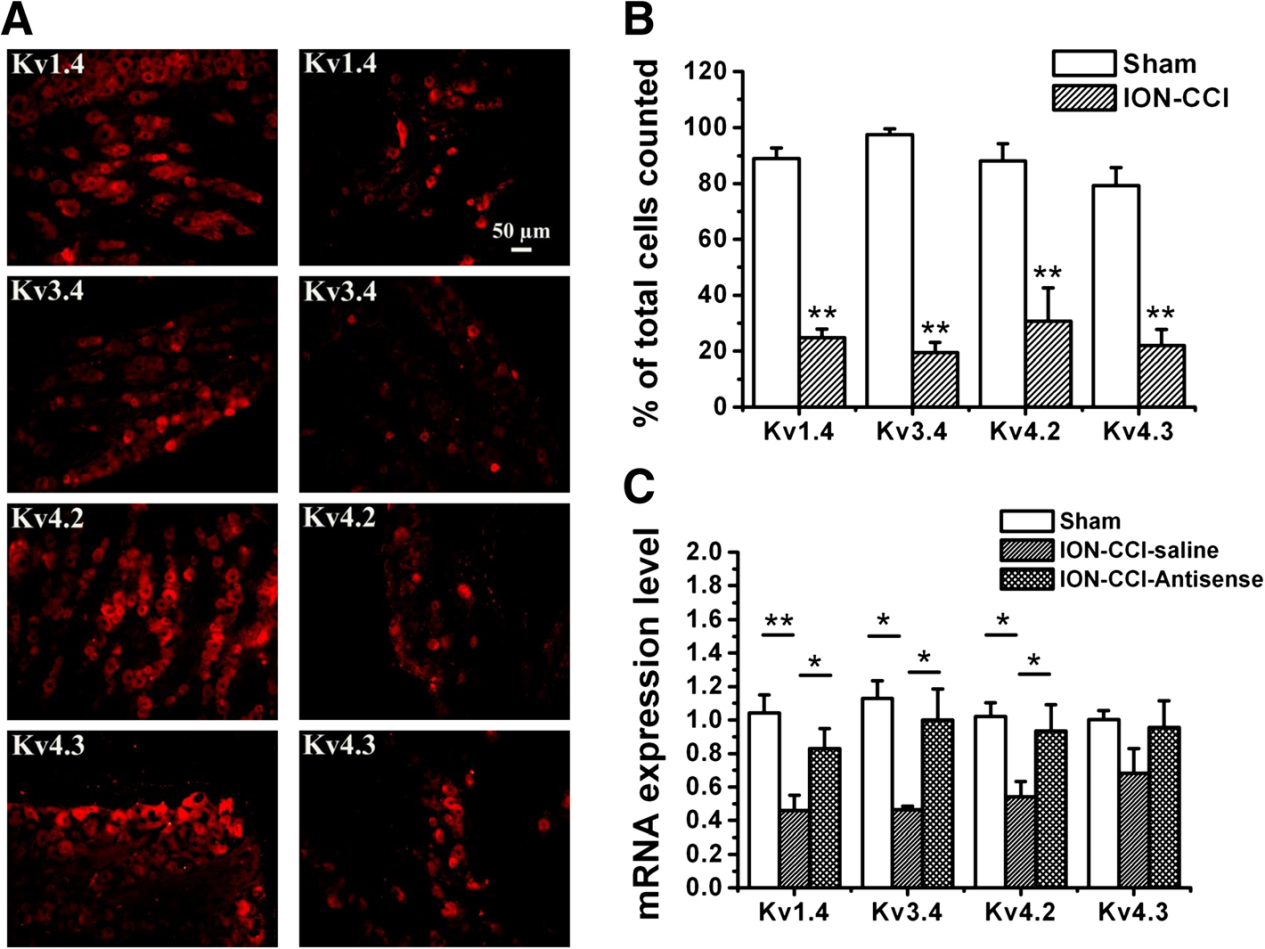
**Difference of I**_**A **_**channel expression in TG between sham and ION-CCI rats. (A)** Double-immunostaining for P2Y_2_ receptors and Kv1.4 or Kv3.4 or Kv4.2 or Kv4.3 on TG neurons in sham and ION-CCI sections, respectively. **(B)** Percentages of numbers of Kv1.4, Kv3.4, Kv4.2 and Kv4.3 subunits in P2Y_2_ receptor-positive neurons are significantly decreased in TG from ION-CCI rats compared with sham rats. n = 4 rats, **, *p* < 0.01). **(C)** Changes in the mRNA levels of I_A_ subunits in TG after P2Y_2_ receptor antisense oligodeoxynucleotides treatment. The mRNA levels of Kv1.4, Kv3.4 and Kv4.2 were significantly decreased in the saline group of ION-CCI rats compared with the sham rats. They were reversed after P2Y_2_ receptor antisense oligodeoxynucleotides treatment. n = 5-9 rats, *, *p* < 0.05, **p < 0.01 compared with saline groups. There was no difference in the levels of Kv4.3 mRNA among the groups. n = 6-8 rats, *p* > 0.05.

To determine whether I_A_-related subunits, Kv1.4, Kv3.4, Kv4.2 and Kv4.3, were changed after ION-CCI, double-immunofluorescence staining for P2Y_2_ receptors and Kv1.4 or Kv3.4 or Kv4.2 or Kv4.3 was performed on TG neurons in sham and ION-CCI groups. In the sham group, 272 out of 301 (90.4%) P2Y_2_ receptor-positive neurons were Kv1.4 positive; 302 out of 307 (98.1%) P2Y_2_ receptor-positive neurons were Kv3.4 positive; 274 out of 325 (84.3%) P2Y_2_ receptor-positive neurons were Kv4.2 positive; and 159 out of 201 (79.1%) P2Y_2_ receptor-positive neurons were Kv4.3 positive. In the ION-CCI group, 42 out of 180 (23.3%) P2Y_2_ receptor-positive neurons were Kv1.4 positive; 42 out of 245 (17.1%) P2Y_2_ receptor-positive neurons were Kv3.4 positive; 66 out of 302 (21.9%) P2Y_2_ receptor-positive neurons were Kv4.2 positive; and 31 out of 166 (18.7%) P2Y_2_ receptor-positive neurons were Kv4.3 positive (Figure 6A). The number of Kv1.4, Kv3.4, Kv4.2 and Kv4.3 subunits on P2Y_2_ receptor-positive TG neurons was significantly decreased in the ION-CCI group compared with that in the sham group (Figure 6B, *p* <0.01, *p* <0.01, *p* <0.01, *p* <0.01). Furthermore, the expression of Kv1.4, Kv3.4 and Kv4.2 in TG neurons was significantly decreased in ION-CCI group compared with that in sham group (Figure 6B, *p* <0.01, *p* <0.05, *p* <0.05). Treatment with P2Y_2_ receptor AS-ODN (15 μg/50 μl) significantly reversed the reduction of Kv1.4, Kv3.4 and Kv4.2 mRNA expression after ION-CCI (Figure 6C, *p* <0.05, *p* <0.05, *p* <0.05). However, the expression of Kv4.3 mRNA was not different among three groups (Figure 6C, *p* > 0.05).

#### Activation of P2Y_2_ receptors mediates an inhibition of I_A_ channels through ERK pathways on small-diameter TG neurons in control rats

**Figure 7:**
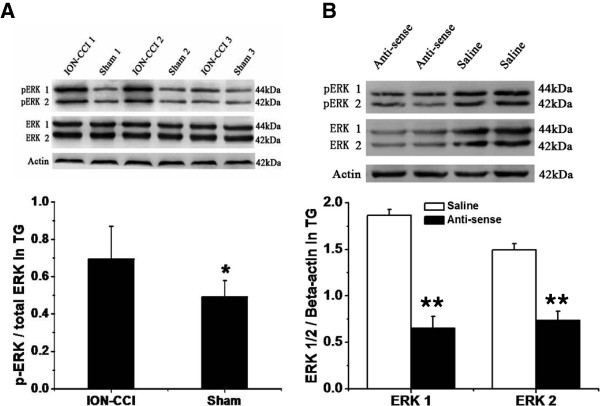
**Role of ERK pathway in activation of P2Y**_**2 **_**receptors mediates an inhibition of I**_**A **_**channels on small-diameter TG neurons in control rats. (A)** Comparison of the phosphorylation of ERK1/2 in TG from sham and ION-CCI rats. Western blot results showed that the level of ERK1/2 phosphorylation was significantly increased in the ipsilateral TG after ION-CCI, compared with that from the sham group. n = 5 for each group *, *p* < 0.05. **(B)** In TG from ION-CCI rats, treatment with P2Y_2_ receptor antisense oligodeoxynucleotides (15 μg/50 μl) significantly decreased the expression of ERK protein in TG. n = 5 for each group, **, *p* < 0.01.

**Figure 8:**
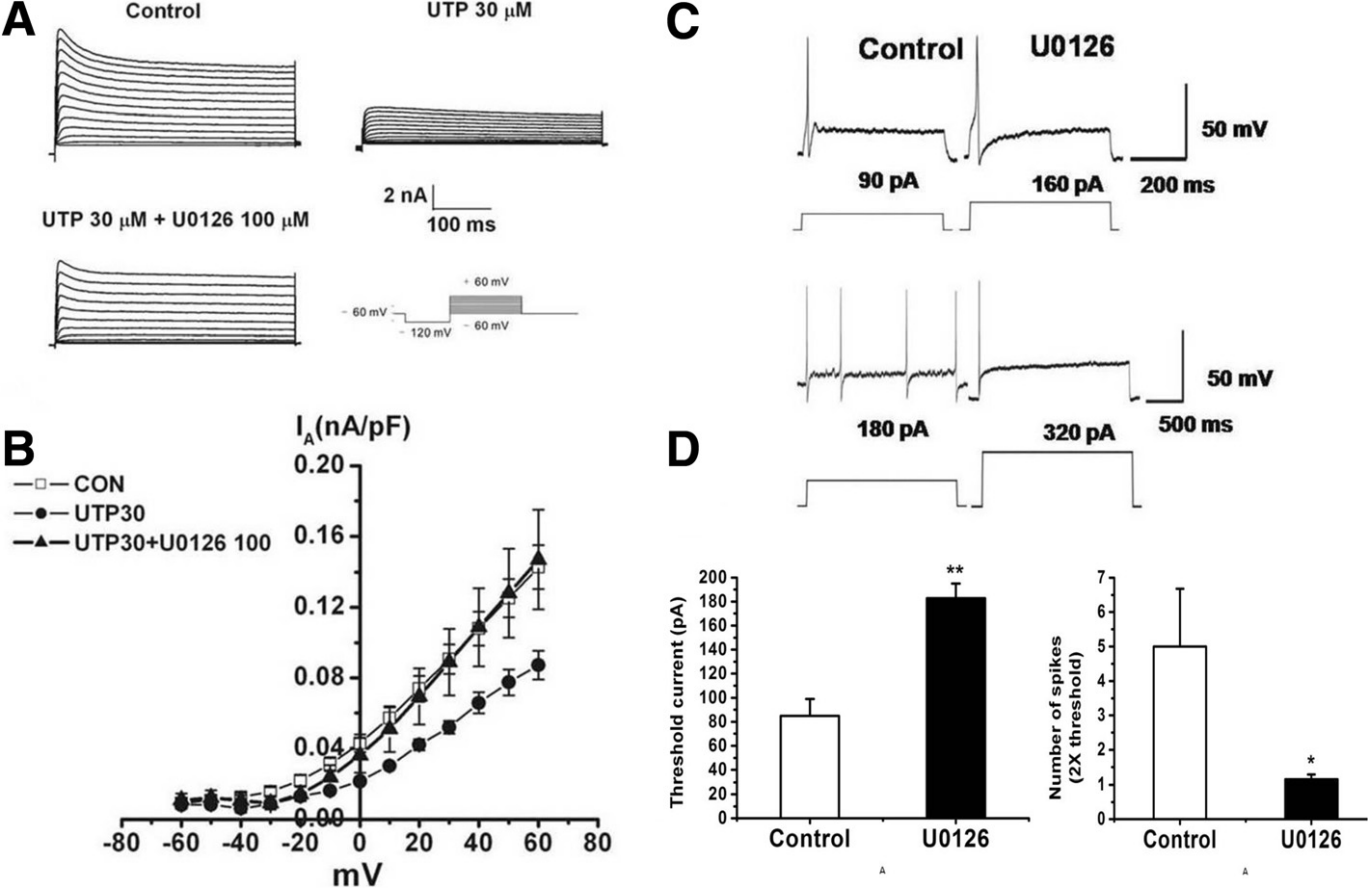
**Effects of UTP and U0126 on I**_**A **_**and action potentials of small-diameter TG neurons in control rats. (A)** I_A_ was initiated via a prepulse (100 ms) of −120 mV and test pulses (400 ms) from −60 to +60 mV in a 10 mV step. Original traces showing that the application of 30 μM UTP reduced I_A_. Suppression of the mean peak amplitudes of I_*A*_ seen after UTP application was antagonized by the presence of U0126 100 μM. **(B)** Current–voltage relationship for the effect of UTP (30 μM) and co-application with U0126 (100 μM) on I_A_. The mean peak amplitude of I_*A*_ was reversed to 0.15 ± 0.03 nA in the U0126 group, which was significantly different from that of the UTP group (Con: 0.14 ± 0.01 nA, n = 20; U0126: 0.15 ± 0.03 nA, n =11, UTP: 0.09 ± 0.01 nA, n = 20, *p* < 0.05 vs U0126). **(C)** Original traces of action potentials during intracellular current injection in ION-CCI TG neurons. **(D)** Mean threshold currents (left panel, n = 8 neurons) and mean number of spikes (right panel, n = 7 neurons) in the presence and absence of U0126 (100 μM) treatment for 16 h. The depolarizing step current amplitude is twice that of the threshold, *, *p* < 0.05, **, *p* < 0.01 vs control.

Western blot results showed that the level of ERK1/2 phosphorylation was significantly increased in the ipsilateral TG after ION-CCI compared with that from the sham groups (Figure 7A, n = 5 for each group, *p* <0.05). It has been reported that ERK activation contributes to changes in membrane excitability as a result of direct or indirect phosphorylation of kinases, key receptors, and ion channels [30]. ERK inhibitors (U0126 or PD98059) enhance A-type potassium currents in dorsal horn neurons of the spinal cord [40,41], indicating the possible modulation of ERK in potassium channels. We therefore tested whether the presence of an ERK inhibitor, U0126, would modulate the effect of UTP on trigeminal pain perception. In TG from ION-CCI rats, treatment with P2Y_2_ receptor AS-ODN (15 μg/50 μl) significantly decreased ERK expression at the protein level (Figure 7B, n = 5 for each group, *p* < 0.01). The inhibitory effect of UTP on I_A_ was significantly reversed (Figure 8A). The mean peak amplitude of was reversed to 0.15 ± 0.03 nA in the U0126 group (100 μM, n = 11), which was significantly different from that of the UTP group (30 μM, 0.09 ± 0.01 nA, n = 20, *p* < 0.05, Figure 8B). Further, in the cultured ION-CCI TG neurons, in the presence of U0126 (100 μM), the mean threshold intensities for evoking action potentials was significantly increased (control: 85 ± 14 pA; U0126: 182.9 ± 12.1 pA; n = 8, Figure 8C,D, *p* < 0.01 vs control), while the number of action potentials was significantly decreased (Figure 8C,D, n = 7, *p* < 0.05 vs control).

## Discussion

Four major findings arise from this study, 1) P2Y_2_ receptors and Kv1.4, Kv3.4, Kv4.2 or Kv4.3 channels were co-expressed in rat TG neurons. The expression of Kv1.4, Kv3.4, Kv4.2 or Kv4.3 on P2Y_2_ receptor-positive TG neurons was significantly decreased after ION-CCI; 2) UTP application enhanced the excitability of control TG neurons and depressed the I_A_ currents, which could be reversed by suramin; 3) activation of P2Y_2_ receptors down-regulated mRNA expression and function of Kv1.4, Kv3.4, Kv4.2 and Kv4.3 on TG neurons in control rats; 4) after the expression of P2Y_2_ receptors was suppressed by AS-ODN treatment, mechanical allodynia was reduced and mRNA levels of Kv1.4 and Kv3.4 and Kv4.2 were increased in ION-CCI rats. These results provide evidence that the down-regulation of I_A_-related potassium channels by activation of P2Y2Rs in TG neurons potentiates neuronal excitability which then contributes to trigeminal neuropathic pain.

### Activation of P2Y_2_ receptors enhances TG neuron excitability through suppression of I_A_ channels in control rats

Growing evidence indicates that P2X and P2Y receptor-mediated signaling critically contributes to the development and maintenance of neuropathic pain [12,13]. Here, we have demonstrated that activation of P2Y_2_ receptors leads to a significant increase in the excitability of TG neurons. Previous studies have shown that an increase in membrane excitability in DRG neurons was a cellular-correlate of enhanced nociceptive behavior [18,42,43]. ATP is a non-selective agonist for several ionotropic P2X and metabotropic P2Y receptor subtypes [44]. Usually, ATP released from healthy cells plays a physiological role [45]. In pathological conditions, ATP release can be evoked from sensory neurons and it produces fast excitatory potentials in DRG cells [46]. Thus, endogenously released ATP from damaged cells [47] may contribute to the ectopic firing of Aβ and Aδ neurons and lead to the development of allodynia [48]. In the present study, ATP caused a large decrease in the mean threshold intensities for evoking action potentials and a significant increase in the mean number of spikes in control TG neurons, which is consistent with a previous study [3]. Although there are (controversial) conflicting outcomes following the use of UTP via P2Y_2_ receptors for neuropathic pain [16-19,48], this study demonstrated that UTP caused a large decrease in the mean threshold intensities for evoking action potentials and a significant increase in the mean number of spikes in control TG neurons. UTP has a similar effect on sensory neurons and thus plays a key role in the development of mechanical allodynia [48]. These results suggest that nucleotides enhance the excitability of TG neurons, probably via both P2Y_2_ and P2Y_4_ receptors, because UTP is a P2Y_2_/P2Y_4_ receptor agonist.

Kv channels are crucial in the control of neuronal excitability, and their down-regulation leads to an increase of neuronal excitability [26,49,50]. Homomeric Kv1.4 channels predominate in Aδ and C fibers arising from small-diameter DRG neurons [28]. Morgan et al. [51] reported that Kvl.4 and Kv4.2, which form transient (A-type) K^+^ channels, may regulate synaptic transmission via presynaptic or postsynaptic mechanisms, respectively. The present electrophysiological study found that UTP mediated a functional inhibition of I_A_ channels in FG-labeled small-diameter TG neurons in control rats. UTP-induced depression of I_A_ was blocked by suramin, hence, the P2Y_2_ nucleotide receptor must have contributed for the following reasons: (1) UTP, a P2Y_2_/P2Y_4_ receptor agonist enhanced the excitability of TG neurons and inhibited I_A_. (2) ATP and UTP were about equipotent as observed for rat P2Y_2_ and P2Y_4_ receptors [52]. (3) Suramin, which is a relatively selective antagonist of P2Y_2_ receptors reversed the UTP-induced inhibition of I_A_[7,53]. (4) α,β-meATP, a P2X_3_ and P2X_2/3_ receptor agonist and 2-MeSADP, a P2Y_1_ receptor agonist did not inhibit I_A_. Thus activation of P2Y_2_ receptors enhanced excitability of TG neurons probably by suppressing I_A_.

Inhibition of I_A_ can increase the firing frequency and broaden the action potential leading to increased Ca^2+^ influx and neurotransmitter release [33,50,54]. The Kv subunits, Kv1.4, Kv3.4, Kv4.2, and Kv4.3, could be dominant in contributing to I_A_. Kv3.4 was expressed mainly by nociceptive DRG neurons where Kv4.3 appeared selectively in the soma of a subset of non-peptidergic nociceptive DRG neurons, and reduced expression of Kv4.3 in pain-sensing neurons may induce neuropathic pain [26]. Hu et al. [55] found that genetic elimination of Kv4.2 reduced I_A_ and increased excitability of dorsal horn neurons. The expression of mRNA for Kv1.4, Kv3.4, Kv4.2, and Kv4.3 was markedly reduced in diabetic neuropathic rats [27]. Combined with our electrophysiological data, the down-regulation of I_A_ subunits, including mRNA for Kv1.4, Kv3.4, Kv4.2, and Kv4.3, after application of UTP, could account for the reduced I_A_ observed in UTP-incubated small diameter TG neurons from control rats. Suramin reversed the UTP-induced effect on TG neurons in control rats, further suggesting that P2Y_2_ receptors were involved.

### The involvement of P2Y_2_ receptors in mechanical allodynia in ION-CCI rats

In this study, we found the expression of Kv1.4, Kv3.4, Kv4.2 and Kv4.3 on P2Y_2_ receptor-positive TG neurons significantly decreased after ION-CCI compared with those in the sham group. Expression of P2Y_2_ receptors, Kv1.4, Kv3.4, Kv4.2 and Kv4.3 was significantly reduced in ION-CCI rats. These data imply that I_A_ channel expression levels of nociceptors and nerve ligation-induced neuropathic pain could be closely related.

The present study showed that activation of P2Y_2_ receptors could suppress I_A_ channels in control rats, which might be one of the mechanisms of hyperexcitability of TG neurons after UTP application. We hypothesized that block of P2Y_2_ receptors could relieve trigeminal neuropathic pain. Firstly, we confirmed that suramin led to a time- and dose-dependent decrease in pain-related behavior of ION-CCI rats. Some similar observations were reported concerning the analgesic effects of suramin in animal pain models [56,57]. Because suramin is an antagonist of P2Y receptors except P2Y_4_ and P2Y_6_ receptors [57], the results suggest that P2Y_1_, P2Y_2_, P2Y_11_, P2Y_13_ and P2Y_14_ receptors could affect pain-related behavior in ION-CCI rats. Considering the effect of UTP in control rats, we concluded that P2Y_2_ receptors were probably involved in ION-CCI-induced pain behavior. Secondly, injection of P2Y_2_ receptor AS-ODN significantly alleviated mechanical hypersensitivity 6 h after injection, which remained until 120 h. The results further support that block of P2Y_2_ receptors could relieve trigeminal neuropathic pain.

To test whether there is a correlation between mechanical sensitivity and I_A_ channel expression, we measured the mRNA levels of the I_A_-related potassium channels, Kv1.4, Kv3.4, Kv4.2 and Kv4.3, in TG neurons before and 36 h after P2Y_2_ receptor AS-ODN treatment. The mRNA expressions of Kv1.4, Kv3.4 and Kv4.2 subunits were markedly reduced after ION-CCI, which were then reversed after selective knockdown of P2Y_2_ receptor gene expression. It has been reported that there is a close relationship between P2Y and Kv channels. ATP and UTP reversibly inhibited the voltage-gated K^+^ currents in *Xenopus* embryo spinal neurons [58]. KCNQ1/KCNE1 K^+^ channels and P2Y_4_ receptors are co-expressed from the time of birth in the apical membrane of rat strial marginal cells [59]. Purinergic P2Y agonists suppress M currents (I_M_), which are generated by Kv7 [18,60]. Our results suggest that activation of P2Y_2_ receptors could result in the development of mechanical hypersensitivity, a major symptom of neuropathic pain, which could be as a result of the suppression of the mRNA expression of Kv1.4, Kv3.4 and Kv4.2 subunits.

In the present study, the expressions of Kv4.3 in mRNA and protein levels were decreased after application of UTP in cultured TG neurons from control rats (Figure 6A,B), but did not change in TG after ION-CCI (Figure 6C). This may be because: (1) Kv4.3 channels were not prominent in the development of allodynia in ION-CCI rats; and (2) an increase of Kv4.3 channels in glial cells surrounding the neurons in TG compensated for the changes in TG neurons after ION-CCI. Expression of I_A_-related K_V_ channels, such as Kv4.1, in glial cells suggests that glial cells also play an important role in chronic pain [61,62]. Further research of Kv channels on TG glial cells is required to explain how I_A_ channels are involved in trigeminal neuropathic pain.

### ERK1/2 is the downstream kinase for the effect of P2Y_2_ receptors on I_A_ channels

P2Y_2_ receptors are G protein-coupled receptors that usually activate PLC-β via G_αq_, which results in the release of intracellular Ca^2+^ and activation of PKC [63]. These events further activate extracellular signal-regulated kinase (ERK), including ERK1 and ERK2 [64-66]. ERK and Kv4.2 have a functional link at both the cellular and behavioral levels [67]. Phosphorylation of Kv4.2 by PKC enhanced ERK phosphorylation of the channel *in vitro*. These findings suggest the possibility that Kv4.2 is a locus for PKC and ERK cross-talk [68]. Kv4.3 positive neurons also expressed ERK2 and mGluR5, suggesting that Kv4.3 subunits could be involved in pain modulation [69]. In line with the previous report [70], we found that ION-CCI significantly increased the level of ERK1/2 phosphorylation in TGs. Evidence provided in this study further suggests that the inhibition of I_A_ channels through P2Y_2_ receptors is modulated by ERK signaling after ION-CCI. First, I_A_ was significantly inhibited by UTP, which could be reversed when ERK signaling was blocked by U0126. Second, in ION-CCI rats, the expression of ERK in protein level was increased and the mRNA expressions of Kv1.4, Kv3.4 and Kv4.2 subunits were decreased, which were then reversed by P2Y_2_ receptor AS-ODN treatment. A recent study has shown that the PI3K/Akt signaling pathway can be activated by P2Y_2_ receptors [71]. The PI3K/Akt signaling pathway and Kv channels are both involved in the same disease [72]. Although we could not exclude that other pathways contribute to this effect of UTP, the ERK signaling pathway might be one of the downstream pathways for the effect of P2Y_2_ receptors on I_A_ channels, which might contribute to the development of trigeminal neuropathic pain.

In the present study, the effect of UTP on mechanical pain threshold in normal rats started from 10 min, suggesting the pathway without alterations of gene expression. The possibilities could be through facilitating homomeric P2X_2_[19], P2X_3_[19,73], or TRPV1 receptors [16,74]. Further, the long-term effect of UTP (more than 30 min) in pain behavior study and antisense oligodeoxynucleotides effect on ION-CCI rats indicate the alterations of gene expression. Although the underlying mechanisms are not fully understood, inhibition of P2Y_2_ receptors leads to down-regulation of ERK-mediated phosphorylation and increase of the expression of I_A_–related Kv channels in trigeminal ganglion neurons, which might contribute to the clinical treatment of trigeminal neuropathic pain. Taken together, these data suggest that P2Y_2_ receptors on TG might play an important role in initiating and maintaining the allodynia in trigeminal neuropathic pain.

## Abbreviations

UTP: Uridine 5′-triphosphate; ATP: Adenosine 5′-triphosphate; ION-CCI: Chronic constriction nerve injury of the infraorbital nerve; TG: Trigeminal ganglion; AS-ODN: Antisense oligodeoxynucleotides.

## Competing interests

The authors declare that they have no competing interests.

## Authors’ contributions

NL designed and contributed all experiments. NL and ZYL performed pain behavioral tests, immunohistochemical staining, and patch-clamp recording, and analysed these experiments data. LHY performed QT-RT-PCR, and Western-blot and analysed these experiments data. BM and XMD contributed to all experiments design and supervised research. NL wrote the manuscript. GB and BM revised the manuscript. Above all authors contributed read and approved the final manuscript.
